# Placental and cerebral circulation in fetuses of mothers with polycystic ovary syndrome and the effect of Metformin exposure

**DOI:** 10.1186/s12884-025-07866-9

**Published:** 2025-07-10

**Authors:** Emma Nordtvedt, Jörg Kessler, Cathrine Ebbing, Ganesh Acharya, Tone S. Løvvik, Kjell Å. Salvesen, Øyvind Salvesen, Eszter Vanky, Birgitte H. Kahrs

**Affiliations:** 1 Department of Clinical and Molecular Medicine, Norwegian University of Science and Technology (NTNU), Trondheim, Norway; 2 Department of Clinical Science, University of Bergen, Bergen, Norway; 3 Department of Obstetrics and Gynaecology, Haukeland University Hospital, Bergen, Norway; 4 Department of Clinical Science, Intervention and Technology, Karolinska Institutet, Stockholm, Sweden; 5 Centre for Fetal Medicine, Karolinska University Hospital, Stockholm, Sweden; 6 Women’s Health and Perinatology Research group, Department of Clinical Medicine, UiT– The Arctic University of Norway, Tromsø, Norway; 7 Department of Obstetrics and Gynaecology, St. Olavs hospital, Trondheim University Hospital, Trondheim, Norway; 8 Department of Public Health and Nursing, Faculty of Medicine and Health Sciences, Norwegian University of Science and Technology, Trondheim, Norway

**Keywords:** Polycystic ovary syndrome, Metformin, Cerebral blood flow, Fetal doppler measurements, Umbilical artery, Cerebroplacental ratio, Middle cerebral artery

## Abstract

**Background:**

Women with polycystic ovary syndrome (PCOS) have an increased risk of pregnancy complications and fetal growth restriction is reported. Increased head size has been reported in PCOS-offspring exposed to intrauterine metformin. We aimed to explore whether maternal PCOS-status, and the use of metformin, during pregnancy affect fetal cerebral and placental circulation.

**Methods:**

The Pilot (2000-2003) and the PregMet2 (2012-2017) studies are randomized controlled trials where women with PCOS were randomized to metformin or placebo in pregnancy. Individually pooled data from Doppler examinations at gestational week 32 from these RCT’s were included in a post-hoc analysis of totally 64 participants. The pulsatility index (PI) z-scores of the fetal middle cerebral artery (MCA), umbilical artery (UmbA), and the cerebroplacental ratio (CPR) were compared between the metformin and placebo treated groups with a two-sample t-test. The PI z-scores of the women with PCOS were compared to low-risk reference populations of pregnant women. The PI z-scores were correlated with maternal BMI at inclusion, androgen levels measured at gestational week 32, percentage gestational weight gain from first trimester to gestational week 32 and offspring head circumference.

**Results:**

There was no significant difference in PI z-scores of fetal MCA, UmbA, and CPR between metformin and placebo treated women with PCOS, or between women with PCOS compared to low-risk reference populations. Maternal BMI, androgen levels, percentage gestational weight gain, and offspring head circumference did not correlate significantly with PI z-scores of fetal MCA, UmbA or CPR.

**Conclusion:**

Maternal PCOS and metformin exposure during pregnancy had no significant impact on placental and fetal cerebral pulsatility indices. The reported fetal growth restriction in PCOS-offspring and the larger head size in metformin exposed fetuses may not be explained by altered umbilical and cerebral pulsatility indices, or their relationship. These findings support the current clinical practice that fetoplacental Doppler assessment in PCOS pregnancies should follow standard obstetric indications.

**Trial registration:**

The Pilot study was performed before trial registrations became mandatory and the trial was retrogradely registered on the 17th of August 2017. ClinicalTrials.gov Identifier: NCT03259919. The PregMet 2 study was registered on the 26^th^ of April 2012 at ClinicalTrials.gov Identifier: NCT01587378. The CircMet study was a sub-study of the PregMet 2 study.

**Supplementary Information:**

The online version contains supplementary material available at 10.1186/s12884-025-07866-9.

## Introduction

Polycystic ovary syndrome (PCOS) is the most common endocrine disorder in women, with a prevalence of 10–18% in the general population [1, 2]. PCOS affects women through the entire lifespan and has negative impact on both reproductive, metabolic, cardiovascular, and psychologic health [3]. Pregnancy complications such as miscarriage, preterm delivery, gestational diabetes mellitus, and preeclampsia are 2-4-fold increased among women diagnosed with PCOS [4]. Newborns of women with PCOS compared to control women are more often growth restricted [5].

Ultrasound Doppler measurement of fetal and placental circulation is a powerful tool for the surveillance of fetal wellbeing, guide clinical decision-making and predict prognosis, and is used in monitoring complicated and high-risk pregnancies [6]. Despite the high prevalence of pregnancy complications and co-morbidities in women with PCOS, studies on Doppler sonographic evaluation of fetal and placental circulation in PCOS pregnancies are few.

Previously, we have demonstrated that metformin prevents late miscarriage and preterm delivery in pregnant women with PCOS. Metformin also reduced weight gain in pregnancy but did not prevent the development of gestational diabetes mellitus in these women [7]. We found that metformin exposure during fetal life resulted in larger head circumference of the newborn. Among eight-year-old children, metformin exposure during fetal life resulted in higher BMI, more central adiposity, and more obesity [8–10].

In a small study metformin, compared to placebo, did not affect mean IQ of the exposed children, but more children exposed to metformin during pregnancy had borderline intellectual function (IQ-score < 85) [11].

We have also demonstrated that uterine artery pulsatility index (PI) did not differ between pregnant women with PCOS and healthy controls in the first and second trimester [12, 13]. While metformin did not alter uterine artery PI in pregnant women with PCOS, high fasting glucose levels, gestational diabetes mellitus, hypertension, and preeclampsia correlated to increased PI suggesting reduced blood distribution to the placenta [13, 14].

Maternal PCOS status and metformin exposure seem to affect both somatic and psychologic health of the offspring [4, 7–9, 11], but knowledge about the underlying mechanisms is lacking. We hypothesized that maternal PCOS and metformin exposure modifies cerebral blood flow in the fetus as a sign of redistribution due to metabolic stress.

The primary aims of this study were to compare fetal cerebral and placental circulation in pregnancies of women with PCOS treated with metformin versus placebo at gestational week 32. The total group of women with PCOS was compared to low-risk reference populations. A secondary objective was to explore the potential effect of maternal androgen levels, BMI, gestational weight gain, and offspring head circumference on the fetal cerebral and placental circulation.

## Materials and methods

This study is an individually pooled post-hoc analysis of sub-groups originating from two double blinded, randomized controlled trials (RCT); the Pilot study and the PregMet 2.

study [7, 15]. These studies compared the use of metformin, an insulin-resistance lowering drug, to placebo during pregnancy in women with PCOS.

Inclusion criteria were women diagnosed with PCOS according to the Rotterdam criteria [3], age between 18 and 45 years, and a first trimester pregnancy with a single viable fetus confirmed with ultrasound. The participants received counselling on lifestyle and diet according to national guidelines. Average adherence to study medication was 85% in both the Pilot study and the PregMet 2 study [7, 15].

The pilot study, was a single centre RCT, aimed to explore the effect of metformin use during pregnancy in women with PCOS, by investigating maternal androgen levels, pregnancy outcomes and trans-placental passage of metformin [15, 16]. The study was conducted at the Regional Hospital in Trondheim in 2000–2003, now named as St. Olav University Hospital. In total, 40 participants were included. After informed written consent participants were randomized to metformin or placebo. Treatment with study medication started in the first trimester. The dosage was 850 mg metformin, or placebo, twice daily until delivery. Participants were examined with Doppler ultrasound at gestational week 32. Doppler measurements were performed with Vingmed System 5 (Vingmed, Horten, Norway) equipped with a 6.5-MHz transvaginal transducer and a 3.5-MHz transabdominal transducer used with a 125-Hz high-pass filter to eliminate signals from slowly moving tissues. The same investigator (KÅS), who was blinded to the treatment allocation, conducted all the examinations [17].

The PregMet 2 study, a Nordic multicentre RCT, aimed to explore whether metformin reduced late miscarriages and preterm births in women with PCOS [7]. The study was conducted in 2012–2017. In total, 487 women were included. The participants gave informed written consent and were randomized to metformin or placebo. Treatment with 1000 mg metformin, or placebo, twice daily was started shortly after inclusion, during first.

trimester [7]. At Haukeland University Hospital in Bergen the women participating in the PregMet 2 study were invited to join a sub-study– the CircMet study. These women gave separate written consent and were scheduled for an extended ultrasound examination at gestational week 32. Doppler measurements were performed with Voluson E6 and E8 machines (2–5 MHz curved linear abdominal transducer). All Doppler examinations were according to guidelines [6]. A single operator (JK) conducted all the examinations and was blinded to the treatment allocation [18].

The low-risk reference populations in the present study are from works of Ebbing et al. [19]. and Acharya et al. [20]. Both longitudinal studies recruited healthy women with a normal 2nd trimester routine ultrasound scan and without previous pregnancy complications. Ebbing et al. recruited 161 and Acharya et al. included 130 women with low-risk singleton.

pregnancies [19, 20].

As a part of the PregMet 2 study, the study participants provided serum samples after an overnight fasting at gestational week 32. All serum samples were drawn from the antecubital vein in non-heparinized tubes and stored at −80^°^ C in 2 to 6 years before being analysed. Androstenedione (A4) and testosterone (T) were analysed by liquid chromatography mass spectrometry. Sex-hormone binding globulin (SHBG) was determined by immunoassay on a Roche Cobas e602 system (Roche Diagnostics, Mannheim Germany). All hormones were analysed at the Department of Biochemistry, Lillebelt hospital, Denmark. Free testosterone index was calculated as (T/SHBG) x 100% ^(10)^. Data on maternal weight, height, BMI, and neonate head circumference were collected as part of the original studies, the Pilot study and the PregMet 2 study [7, 15].

**Fig. 1:**
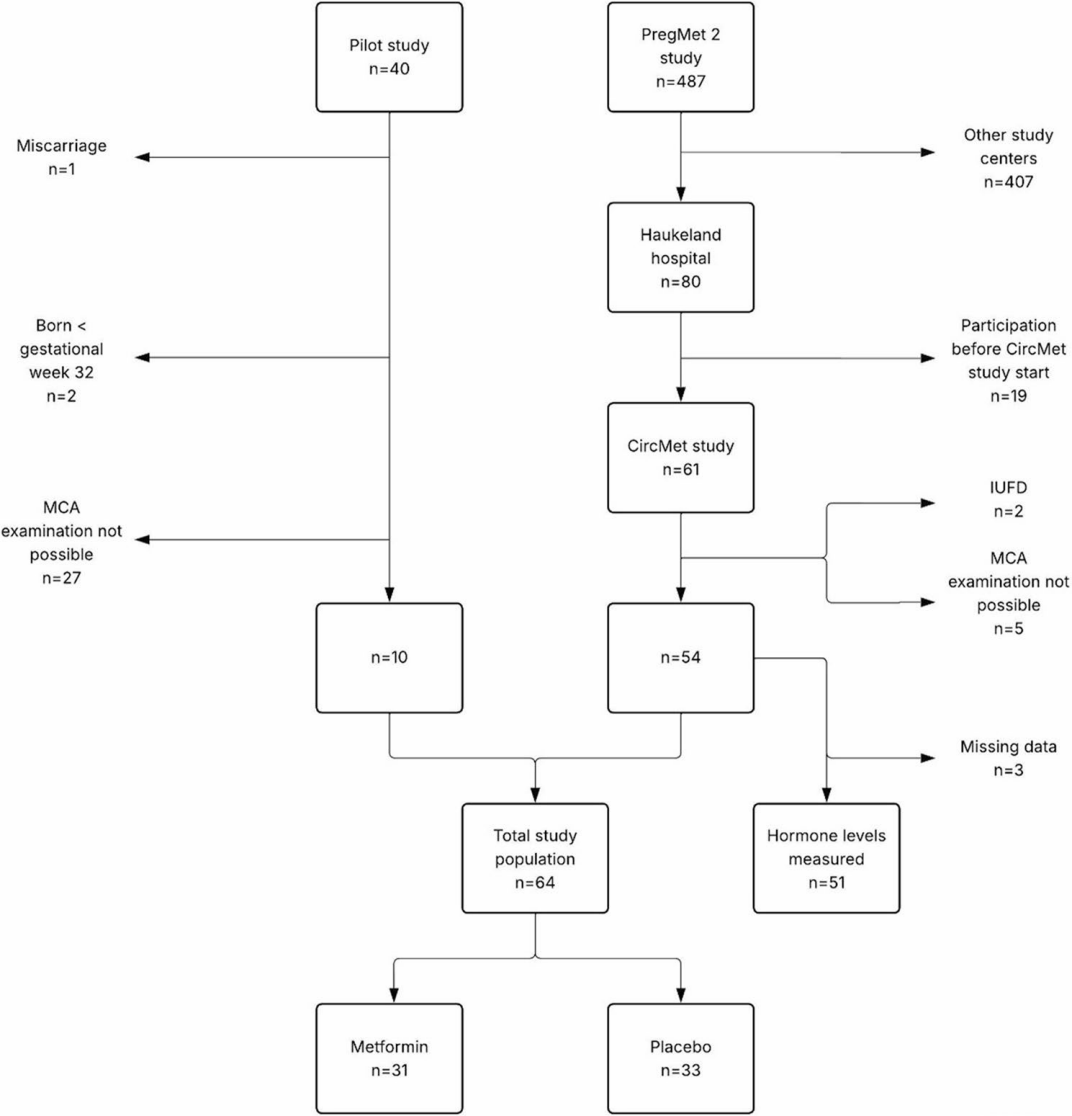
Flow chart of women with polycystic ovary syndrome (PCOS) treated with metformin or placebo in the Pilot and the PregMet 2 study included in the individually pooled post-hoc analyses of this study

A total of 64 women diagnosed with PCOS are included in this study, 10 from the Pilot study and 54 from the PregMet 2 study (Fig. 1). Only the participants with an available middle cerebral artery (MCA) PI examination were included for the per-protocol analyses in this study. Missing data for the other main endpoints and secondary endpoints were handled with pairwise deletion for the analyses.

Main endpoints of the present study are PI z-score of the MCA, umbilical artery (UmbA) and the cerebroplacental ratio (CPR), measured with Doppler ultrasound during gestational.

week 32. PI z-score measurements are used to adjust for gestational age. The reference population used to calculate the reference ranges for the z-scores for MCA PI and CPR were the cohort of Ebbing et al. from Haukeland University Hospital in Bergen [19], while the reference ranges for z-score of the UmbA PI was based on the material of Acharya et al. from the University Hospital of North-Norway in Tromsø [20]. Comparison analyses were performed between the PI z-scores in the metformin and the placebo groups.

As a secondary endpoint, we investigated the correlation between PI z-scores and maternal androgens in a non-randomized analysis for the entire group of women with PCOS. Androstenedione (A4), testosterone, sex-hormone binding globulin (SHBG), free testosterone index (FTI), maternal BMI at inclusion, percentage weight gain from inclusion to gestational week 32, and z-score of neonate head circumference at birth, were correlated to the z-score of MCA PI, UmbA PI and CPR. Fetal growth and offspring anthropometric variables for the Pilot and the PregMet 2 study, have been published by Hjorth-Hansen et al. [8] and Nilsen et al. [10].

### Statistical analyses

In this study IBM SPSS Statistics version 27 software was used for statistical analysis. Main endpoints were analysed with a two-sample t-test for comparing the PI z-scores of the women with PCOS treated with metformin to the women with PCOS treated with placebo. The total group of women with PCOS were compared to the low-risk reference populations, by a one sample t-test for comparing total mean values of PI z-scores. Effect sizes were evaluated by 95% confidence intervals of the group differences. The secondary endpoints were analysed using Pearson’s correlation coefficient with a two-tailed significance level to correlate the maternal androgen levels at gestational week 32, BMI at inclusion, percentage gestational weight gain, and z-score of offspring head circumference to the PI z-scores. Effect sizes were evaluated by a two-tailed 95% confidence intervals.

### Ethics statement

The Regional Ethics Committee of Central Norway approved the Pilot study on March the 28th, 2000 (51-2000 REK-Midt). The study was performed before prospective registration of clinical trials became mandatory and the trial was retrospectively registered on ClinicalTrials.gov Identifier: NCT03259919. The PregMet 2 study was approved on 6th October 2011 and CircMet study approved the 7th of October 2013 (REK-midt 2011/1434). ClinicalTrials.gov Identifier: NCT01587378. Approval for further use of data for this study was obtained from the Regional Ethics Committee of Central Norway (REK-reference: 255504). Informed written consent was obtained from each participant before inclusion in all the studies. The declaration of Helsinki was followed throughout all studies. The study adheres to the CONSORT guidelines for reporting clinical trials [21].

## Results

Baseline characteristics are presented in Table 1. The metformin and placebo groups were comparable, except systolic and diastolic blood pressure, being higher (*p* < 0.01) in the metformin group by chance (Table 1).

**Table 1:** Maternal characteristics and pregnancy outcomes

Maternal characteristics		
Mean (SD) or n (%)	**Metformin ** ***n*** ** = 31**	**Placebo ** ***n*** ** = 33**
Age (years)	29 (4)	30 (4)
Weight (kg)	78 (15)	74 (16)
Height (cm)	168 (6)	168 (7)
BMI	27.8 (5.5)	26.0 (6.0)
Systolic BP (mmHg)	111 (11)	100 (14)
Diastolic BP (mmHg)	68 (9)	63 (12)
Hypertension	1 (3.2)	0
Education		
Elementary school	0	0
High school	10 (32)	7 (21)
College	17 (55)	16 (49)
University	4 (13)	10 (30)
Marital status		
Married/co-habitant	31 (100)	31 (94)
Single/other	0	2 (6.0)
Parity		
0	14 (45)	14 (42)
1	14 (45)	13 (39)
2	3 (9.7)	6 (18)
Phenotype PCOS		
HA + OA + PCOM	21 (68)	22 (67)
HA + OA	2 (6.5)	5 (15)
HA + PCOM	3 (9.7)	0
OA + PCOM	5 (16)	6 (18)
Smoking	1 (3.2)	2 (6.1)
**Pregnancy outcomes**		
Gestational diabetes mellitus	12 (39)	7 (21)
Preeclampsia	3 (9.7)	1 (3.0)
Preterm birth (< 37 weeks)	1 (3.2)	1 (3.0)
Apgar score < 7 at 5 min	1 (3.2)	1 (3.0)
Placental weight (g)	651 (145)	688 (169)
Birth weight (g)	3588 (450)	3563 (551)
Birth length (cm)	50.7 (2.3)	51.0 (2.3)
Head circumference (cm)	35.7 (1.5)	35.5 (1.4)

Patient characteristics and pregnancy outcomes of women with polycystic ovary syndrome (PCOS) treated with metformin or placebo during pregnancy in the Pilot and the PregMet 2 study. Blood pressure (BP) was significantly lower than the original study cohorts for both groups. There was a statistically significant difference in systolic blood pressure between the two groups. PCOS phenotype characteristics defined as: hyperandrogenism (HA), oligo-amenoré (OA) and polycystic ovarian morphology (PCOM)

We found no difference in PI z-scores of MCA (95% CI of difference of means [−0.50, 0.47], *p* = 0.950), UmbA ([−0.63, 0.48], *p* = 0.794) and CPR ([−0.49, 0,56], *p* = 0.905), between the metformin and placebo group in women with PCOS. There was no difference in PI z-scores of MCA ([−0.10, 0.38], *p* = 0.230), UmbA ([−0.21, 0.34], *p* = 0.620), and CPR ([−0.19, 0.33], *p* = 0.581) comparing the total PCOS population and the low-risk reference populations (Table 2). Boxplots of the comparative analyses of PI z-scores can be found in the supplementary material.

**Table 2:** Comparison of Doppler pulsatility index z-scores in gestational week 32

	Metformin *n* = 31	Placebo *n* = 33	Mean difference	95% CI of difference	*p*-value
PI of artery	Mean (SD)	Mean (SD)			
**MCA**	2.2 (0.43)	2.2 (0.34)			
z-score	0.14 (1.1)	0.15 (0.85)	−0.02	[−0.50, 0.47]	0.950
**UmbA**	0.93 (0.15)	0.94 (0.17)			
z-score	0.032 (1.0)	0.10 (1.2)	−0.07	[−0.63, 0.48]	0.794
**CPR**	2.4 (0.56)	2.4 (0.52)			
z-score	0.088 (1.1)	0.057 (1.0)	−0.03	[−0.49, 0,56]	0.905
	**PCOS *n* = 64**	**Reference**			
	Mean (SD)	50%-percentile			
**MCA**	2.2 (0.38)	2.1 (0.015)			
z-score	0.14 (0.95)	0		[−0.10, 0.38]	0.230
**UmbA**	0.93 (0.16)	0.90			
z-score	0.069 (1.1)	0		[−0.21, 0.34]	0.620
**CPR**	2.4 (0.53)	2.4 (0.066)			
z-score	0.072 (1.0)	0		[−0.19, 0.33]	0.581

Individually pooled post-hoc analyses of Doppler pulsatility indices of women with polycystic ovary syndrome (PCOS) from the Pilot and PregMet 2 study. All pulsatility indices were measured in gestational week 32. The low-risk reference populations of Ebbing et al. and Acharya et al. were used for calculating z-scores for the pulsatility indices of the middle cerebral artery (MCA), umbilical artery (UmbA), and the cerebroplacental ratio (CPR). Independent samples t-test was used for comparing z-scores of the for women with PCOS treated with metformin (*n* = 31) and placebo (*n* = 33). One sample t-test was used for comparing z-scores for the entire group of women with PCOS to the low-risk reference populations. Effect sizes of difference in z-scores between the groups are calculated by a 95% confidence interval (CI) of difference. There was one missing data for UmbA and CPR in the placebo group, and for these variables in the pooled group of women with PCOS

We found no statistically significant correlations between the PI z-scores of MCA, UmbA, and CPR at gestational week 32 and maternal androgen levels, BMI, gestational weight gain or offspring head circumference in women with PCOS (Table 3).

**Table 3:** Correlation analyses of Doppler pulsatility index z-scores and secondary endpoints in gestational week 32

	**Mean (SD)**		**MCA ***n*=64	**UmbA ***n*=63	**CPR ***n*=63
**Androstendione** (nmol/l) *n*=51	10.9 (5.30)	p	−0.194	−0.026	−0.130
		[95% CI]	[−0.446, 0.086]	[−0.302, 0.254]	[−0.394, 0.154]
		*p*- value	0.172	0.858	0.367
**Testosterone** (nmol/l) *n*=51	3.69 (1.94)	p	−0.222	0.025	−0.178
		[95% CI]	[−0.469, 0.057]	[−0.256, 0.301]	[−0.434, 0.106]
		*p*- value	0.117	0.866	0.217
**SHBG** (nmol/l) *n*=51	455 (141)	p	0.083	−0.046	0.114
		[95% CI]	[−0.197, 0.351]	[−0.320, 0.235]	[−0.170, 0.380]
		*p*- value	0.561	0.752	0.431
**FTI** (%) *n*=51	0.849 (0.464)	p	−0.237	0.008	−0.187
		[95% CI]	[−0.481, 0.042]	[−0.271, 0.285]	[−0.442, 0.096]
		*p*- value	0.095	0.958	0.193
**BMI** (kg/m^2^) *n*=64	26.9 (5.79)	p	−0.065	−0.056	0.009
		[95% CI]	[−0.306, 0.183]	[−0.299, 0.195]	[−0.239, 0.256]
		*p*- value	0.607	0.665	0.945
**Percentage gestational weight gain** (%) *n*=64	11.1 (6.35)	p	−0.030	0.087	−0.101
		[95% CI]	[−0.274, 0.217]	[−0.165, 0.327]	[−0.341, 0,150]
		*p*- value	0.812	0.500	0.429
**Head circumference** (z-score) *n*=63	0.192 (0.936)	p	0.095	0.146	−0.033
		[95% CI]	[−0.157, 0.335]	[−0.108, 0.382]	[−0.280, 0.219]
		*p*- value	0.460	0.257	0.802

Individually pooled post-hoc analyses of Doppler pulsatility indices of women with polycystic ovary syndrome (PCOS) from the Pilot and PregMet 2 study. All pulsatility indices (PI) were measured in gestational week 32. The low-risk reference populations of Ebbing et al. and Acharya et al. were used for calculating z-scores for the PI of the middle cerebral artery (MCA), umbilical artery (UmbA), and the cerebroplacental ratio (CPR). Pearson’s correlation coefficient (r) was calculated for all the PI z-scores and the secondary endpoints. Hormonal levels were measured in gestational week 32, body mass index (BMI) was measured at inclusion, and percentage maternal weight gain from inclusion to gestational week 32. Missing data was handled with pairwise deletion. Missing data: One missing data for the z-scores of the UmbA and the CPR. Hormone levels were only measured for the participants in the PregMet 2 study (*n*=51). Other abbreviations: Sex-hormone binding globuline (SHBG) and Free testosterone index (FTI)

## Discussion

The main findings of the present study were that metformin compared to placebo does not seem to have an impact on MCA, UmbA, and CPR PI z-scores. Also, PCOS-status does not seem to affect MCA, UmbA, and CPR PI z-scores compared to low-risk reference populations. Maternal androgen levels, BMI, gestational weight gain and offspring head circumference did not correlate to MCA, UmbA, and CPR PI z-scores.

Metformin use during pregnancy has been associated with restricted fetal and placental growth [22]. We found no effect of metformin exposure on fetal cerebral and placental circulation. An animal study conducted on sheep, suggests that metformin activate metabolic stress pathways in the fetal hepatocytes, which may contribute to the observed fetal growth restriction [23]. However, a previous study from our group examining the same population found that metformin did not affect fetal liver blood flow [18]. This discrepancy may indicate that metformin primarily influences intracellular mechanisms within the fetal liver, rather than altering fetal blood distribution.

We have also reported that metformin treatment in pregnancy had no effect on uterine artery PI in women with PCOS examined at gestational week 19 [12]. Other studies, however, have reported a vasodilatory effect of metformin on the uterine artery in non-pregnant women with PCOS suggesting that the vascular response to metformin may differ depending on pregnancy status [24, 25].

In previous studies, metformin had no significant effect on maternal blood glucose levels in women with PCOS or the development of gestational diabetes mellitus [26, 27]. However, other research suggests that metformin reduce the placental energy production [28, 29]. Large RCTs found that metformin can reduce maternal weight gain in pregnancy and the incidence of preeclampsia for obese women, but does not appear to effect offspring birth weight or development of gestational diabetes [7, 30].

Further, Hanem et al. [9, 31] found increased BMI at both four and eight years of age in children exposed to metformin in utero, which further support metformin’s metabolic effect on the fetus. These findings suggest that metformin may influence fetal and offspring long-term health modulated through other mechanisms than affecting fetal cerebral and placental circulation in pregnancies of women with PCOS, as indicated by our null findings.

In this study, we found no effect of maternal PCOS on fetal cerebral and placental circulation. Previously, we have reported that maternal PCOS is associated with reduced venous liver blood flow in the fetus [18]. Based on these findings, we speculate that maternal PCOS may exert a greater influence on fetal liver perfusion than on cerebral circulation. Given the central role of the fetal liver in regulating metabolism and growth, this altered blood flow could help explain the metabolic dysfunction and growth disturbances observed in offspring of women with PCOS. These include intrauterine growth restriction [5] and the increased risk of higher weight later in life for the offspring of women with PCOS [9, 32]. Stridsklev et al. [13] found no difference in uterine artery PI, when comparing pregnant women with PCOS to healthy controls. Another study found decreased placental volume for their PCOS group, but also a lower uterine artery PI in first trimester of pregnancy for women with PCOS compared to women without PCOS [33]. This difference in uterine artery PI between women with PCOS and reference groups have also been shown in non-pregnant women [25]. The potential effect of maternal PCOS status on the uterine artery and ductus venosus PI stands in contrast to our findings. Nevertheless, it may also indicate that maternal PCOS have a greater effect on circulation in the uterine artery and the placental growth, than the fetal MCA and the UmbA.

Since there was no difference in the Doppler indices between the metformin-treated group and the placebo-treated group, the correlation analyses of secondary endpoints were run with all women with PCOS independent of randomization. Maternal androgen levels, early pregnancy BMI, and percentage gestational weight gain did not correlate significantly to the fetal MCA and UmbA PI z-scores in our study.

Hyperandrogenic phenotypes of PCOS have been associated with an increased risk of pregnancy complications [34], and has been linked to impaired trophoblast differentiation in early placentation in mice-models [35]. Another animal-model of PCOS, using rats, found an association between hyperandrogenism and insulin resistance with impaired spiral artery remodeling and placentation [36]. Regarding these studies, one would expect a correlation between androgen levels and fetoplacental PI z-scores, which was not found in our analysis. Stridsklev et al. [12] found no correlation between androgen measurements and uterine artery PI in gestational week 19, supporting our findings. Sahin et al. [37] found no statistically significant difference in uterine artery PI in the first trimester, when comparing the different phenotypes of PCOS. Nevertheless, they found a decreased placental volume in the hyperandrogenic phenotypes compared to the non-hyperandrogenic phenotype. This might suggest that elevated androgen levels play a more significant role in fetoplacental circulation earlier in the pregnancy, or through other mechanisms than regulating the placental blood flow.

Rial-Crestelo et al. [38] found a significant association between maternal BMI and the 5th centile of the CPR in healthy pregnant women, suggesting that maternal BMI may be used as a factor for determining cut-off values in clinical decision-making. The present PCOS population had an average BMI of 27 kg/m^2^, which can probably explain that we found no correlations between BMI and CPR. This might suggest that maternal body composition and phenotype has a greater impact on fetoplacental vascular impedance than PCOS-status itself.

Offspring head circumference was not significantly correlated to the z-scores of fetal MCA and UmbA PI or the CPR measured at gestational week 32. Our study group has previously reported that newborns exposed to metformin in-utero had larger head circumference at birth compared to the placebo-exposed newborns. This effect, which was detectable already in-utero, was found in the group of overweight/obese mothers and the mothers with a hyperandrogenic phenotype treated with metformin [8, 10].Our results suggests that redistribution in fetal cerebral circulation might not be one of the underlying mechanisms of the observed increased head size of metformin exposed newborns [8].

The strength of this study is the double blinded RCT design and systematic, structured data collection. We also had access to data from two comparable reference populations. Both treatment groups, and the pooled group of women with PCOS for the analyses of secondary endpoints, had a group size larger than 30, a considered threshold for statistical analyses under the assumptions of the central limit theorem [39]. On the other hand, the limited sample size increases the chance for type II errors and might not be able to detect subtle differences in the endpoints. The post-hoc study design, the limited sample size, and baseline differences in the study groups are obvious limitations. This study provides a snapshot of the PI at gestational week 32. Longitudinal observations of PI development in pregnancy would be preferrable. Also, Doppler examinations may be hampered by abdominal adiposity in women diagnosed PCOS. The Doppler examinations were conducted by two different examinators with different ultrasound machines (Vingmed and Voluson). Although, the examinators were experienced fetal medicine experts, which reduces the chances of errors, different examinators and machines also introduces a potential variation in how the examinations were performed and their results. On the other hand, this also strengthens the external validity. Women included to these two RCTs were mostly of Nordic Caucasian origin, which makes it possibly less applicable to other ethnicities. This homogenous population reduces the external validity.

From a clinical point of view, our findings would suggest that women diagnosed with PCOS should have fetoplacental Doppler examinations for the same indications as the general population of pregnant women. Nevertheless, women with PCOS are a heterogenous group with different characteristics and challenges. Hyperandrogenism, insulin resistance, and maternal obesity are related characteristics of PCOS [40], and may all influence fetoplacental circulation and offspring health in different manners. The potential effect of maternal PCOS phenotype, different BMI groups, and the potential metformin effect for these subgroups on fetal cerebral and placental circulation needs further investigation.

## Conclusion

Maternal PCOS and metformin exposure during pregnancy did not affect fetal cerebral and placental circulation at gestational week 32, suggesting that the reported fetal growth restriction in PCOS-offspring and the larger head size in metformin exposed fetuses may not be explained by altered fetal cerebral and umbilical blood distribution. These findings support the current clinical practice that fetoplacental Doppler assessment in PCOS pregnancies should follow standard obstetric indications.

## Supplementary Information

Supplementary Material 1.

Supplementary Material 2.

## Data Availability

Proposals to use de-identified participant data material from the Pilot study and the PregMet 2 study can be directed to project leader E. Vanky.

## Abbreviations

BMI: Body mass index
PCOS: Polycystic ovary syndrome
PI: Pulsatility index
RCT: Randomized controlled trial
UmbA: Umbilical artery
MCA: Middle cerebral artery
CPR: Cerebroplacental ratio
