# Supplementary figures and images for: Placental and cerebral circulation in fetuses of mothers with polycystic ovary syndrome and the effect of Metformin exposure
Source: BMC Pregnancy Childbirth. 2025 Jul 10;25:749. doi: 10.1186/s12884-025-07866-9 (PMC12243356; doi:10.1186/s12884-025-07866-9)

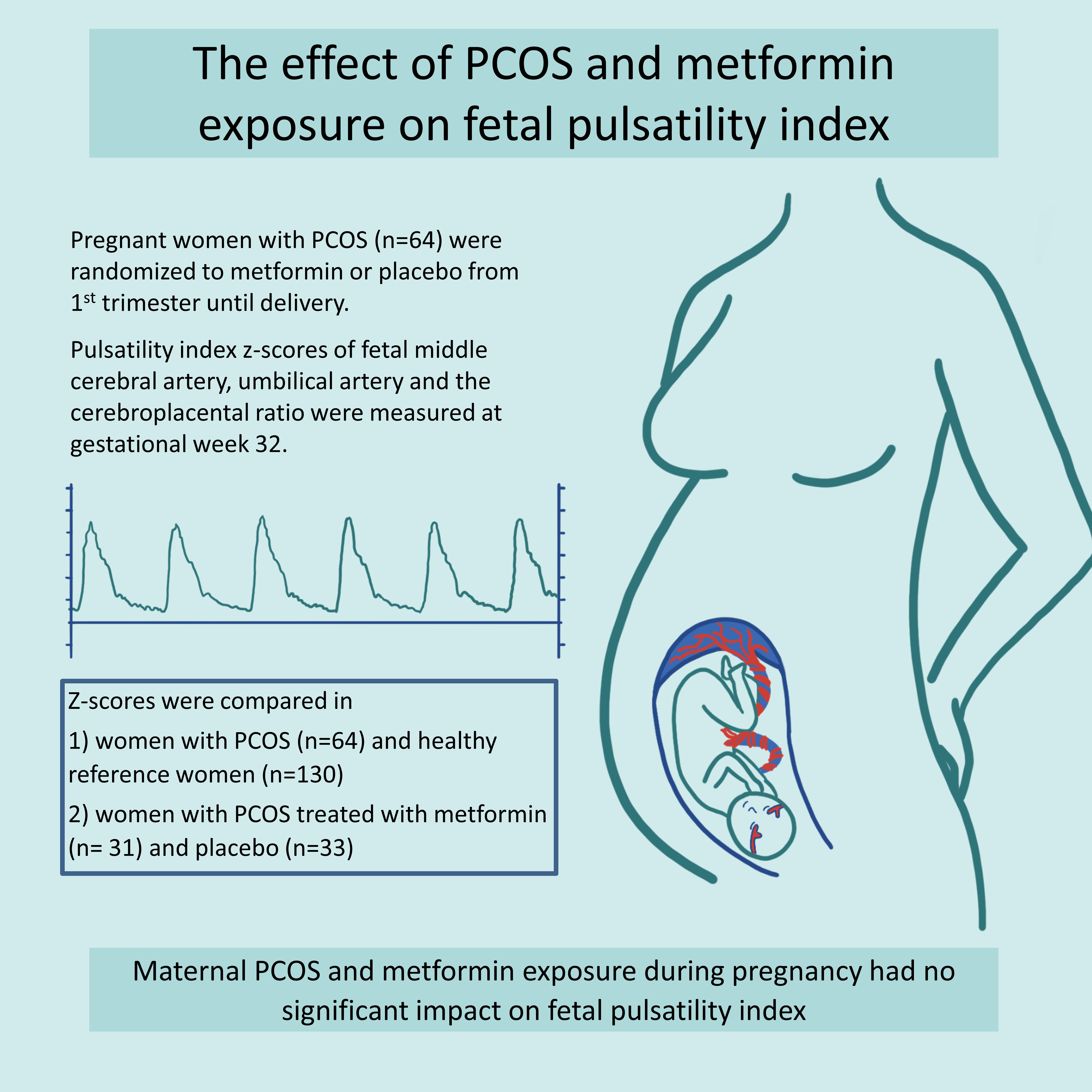

Supplement: Supplementary file 1 — Supplementary Material 1. [file 12884_2025_7866_MOESM1_ESM.jpg]
