# Supplementary material for: Placental and cerebral circulation in fetuses of mothers with polycystic ovary syndrome and the effect of Metformin exposure
Source: BMC Pregnancy Childbirth. 2025 Jul 10;25:749. doi: 10.1186/s12884-025-07866-9 (PMC12243356; doi:10.1186/s12884-025-07866-9)
Supplement: Supplementary file 2 — Supplementary Material 2. [file 12884_2025_7866_MOESM2_ESM.docx]

**Supplementary figures**

Boxplot of middle cerebral artery pulsatility index z-score


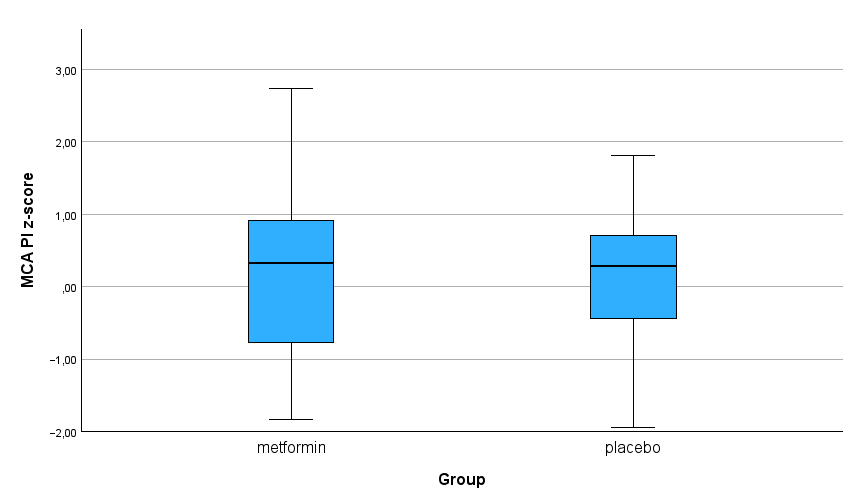


**Figure 2.** Boxplot of middle cerebral artery (MCA) pulsatility index (PI) z-score in the metformin (n=31) and the placebo (n=33) treated groups of women with polycystic ovary syndrome from the Pilot and the PregMet 2 study. Examinations were performed in gestational week 32.

Boxplot of umbilical artery pulsatility index z-score


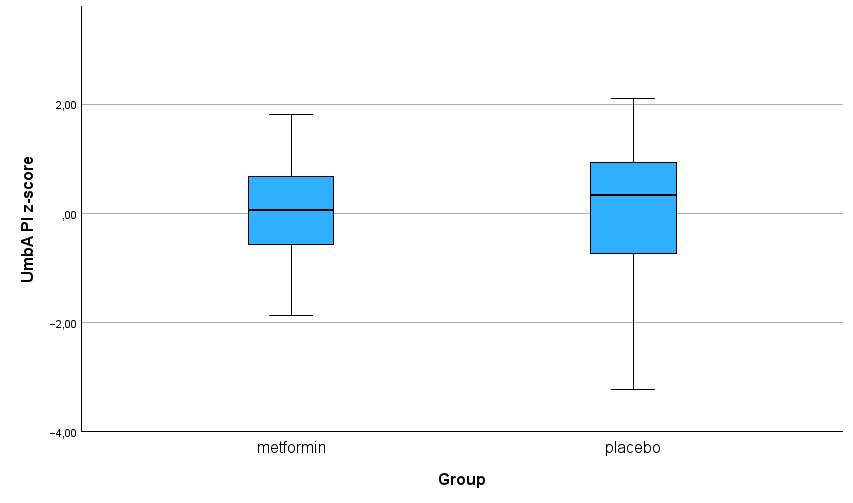


**Figure 3.** Boxplot of umbilical artery (UmbA) pulsatility index (PI) z-score in the metformin (n=31) and the placebo (n=32) treated groups of women with polycystic ovary syndrome from the Pilot and the PregMet 2 study. Examinations were performed in gestational week 32.

Boxplot of cerebroplacental ratio z-score


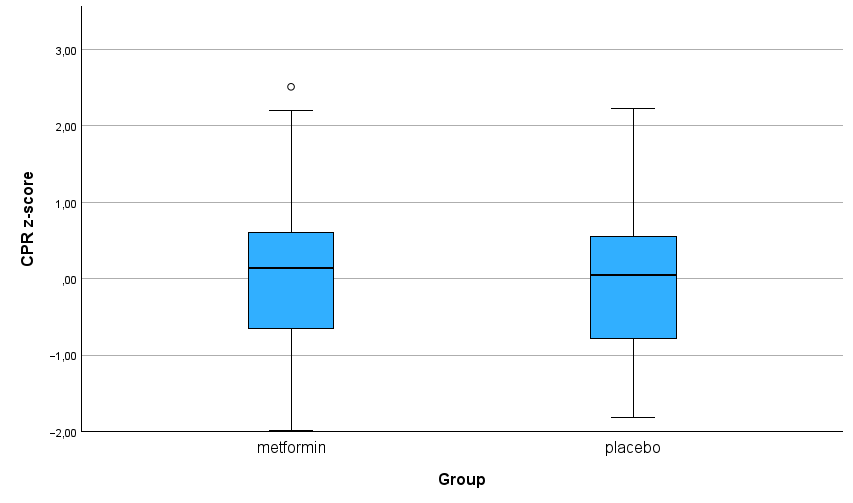


**Figure 4.** Boxplot of cerebroplacental ratio (CPR) z-score in the metformin (n=31) and the placebo (n=32) treated groups of women with polycystic ovary syndrome from the Pilot and the PregMet 2 study. CPR is the ratio of middle cerebral artery pulsatility index to the umbilical artery pulsatility index.
